# Leukocytoclastic Vasculitis: Depiction of the Diagnostic Dilemma

**DOI:** 10.7759/cureus.17462

**Published:** 2021-08-26

**Authors:** Siddharth Bhesania, Karanrajsinh Raol, Chanoa Medina, Sahar Ilyas, Janki Bhesania, Alina Barmanwalla

**Affiliations:** 1 Internal Medicine, Overlook Medical Center, Summit, USA; 2 Internal Medicine, NewYork-Presbyterian Brooklyn Methodist Hospital, Brooklyn, USA; 3 Internal Medicine, Gujarat Medical Education & Research Society (GMERS) Medical College and General Hospital, Gandhinagar, IND; 4 Internal Medicine, White Plains Hospital, White Plains, USA; 5 Internal Medicine, Brigham and Women’s hospital, Boston, USA; 6 Internal medicine, NewYork-Presbyterian Brooklyn Methodist Hospital, Brooklyn, USA

**Keywords:** leukocytoclastic vasculitis (lcv), trimethoprim-sulfamethoxazole (tmp-smx), bactrim ds, vasculitis, corticosteroids

## Abstract

Vasculitis is classified based on the size of the blood vessels involved. Sub-group Leukocytoclastic vasculitis (LCV) refers to small blood vessel inflammation, which involves cutaneous capillaries and venules. To date, there have been myriad primary and secondary probable causes of LCV. Here, we present a case of an 86-year-old male who presented with non-blanchable purpura involving the ankles, knees, and palms. The patient had idiopathic pulmonary fibrosis (IPF), for which he had been on long-term oxygen therapy and chronic corticosteroids. He was recently started on Bactrim DS (trimethoprim-sulfamethoxazole double strength) for prophylaxis of pneumocystis pneumonia. After a meticulous workup, including a skin biopsy, the causative agent of the LCV was established to be Bactrim DS, and the event was likely triggered by superimposed acute stress of sepsis secondary to UTI and bacteremia. There were several diagnostic dilemmas due to the ongoing chronic medical conditions; however, the occurrence of LCV while being on chronic corticosteroids was concerning as it should have prevented such an untoward occurrence. Eventually, the presentation subsided past an increase in the dose of corticosteroids and discontinuation of Bactrim DS. This raises concern regarding either the dose-dependent immunosuppressive effects of corticosteroids or deficits in our current understanding of the mechanism of action. Additionally, it necessitates further exploration into the causes of LCV and a thorough understanding of its pathogenesis.

## Introduction

Leukocytoclastic Vasculitis (LCV) is an inflammatory condition of small-sized blood vessels. The major pathogenesis of which is estimated to be the deposition of immune complexes in the vessel wall of small-sized vessels, including capillaries and venules. LCV can be primary or secondary to an underlying bigger picture of systemic infections, autoimmune and connective tissue disorders, drugs, and tumors [1]. LCV is not confined to any specific age group or sex, provided the susceptibility and the inciting agent can occur in a person of any age group. Drug-induced vasculitis could reach its peak anywhere between 1-3 weeks [2]. Here, we report a case of an elderly male who was on chronic corticosteroids for idiopathic pulmonary fibrosis and developed LCV after initiation of Bactrim DS. This report enhances the necessity to explore the etiologies and the pathogenesis of LCV since the patient was already on the standard treatment for LCV and yet ended up developing the same.

## Case presentation

**Figure 1:**
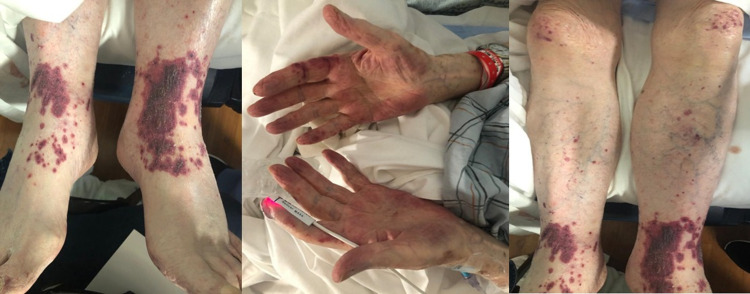
Non-blanchable purpura over bilateral ankles, knees, and palms

An 86-year-old male presented to the emergency room with a 3-week history of a progressive macular, purple-colored rash present over the ankles, which began as discoloration over the left ankle and progressed to involve ankles, knees, and palms bilaterally(Figure 1). Apart from the rash, the patient reported occasional fevers and chills for three days but no other constitutional symptoms. He denied any recent travel, sick contacts, or nature excursions. Past medical history is notable for hypertension, hyperlipidemia, hypothyroidism, gout, benign prostatic hypertrophy (BPH), and idiopathic pulmonary fibrosis (IPF) on 3-4 L home oxygen and chronic moderate dose corticosteroids. Remote history was significant for colon adenocarcinoma (in remission), an episode of deep venous thrombosis (DVT), and abdominal aortic aneurysm s/p repair. Patient-reported compliance with treatment and had regular outpatient follow-ups with his respective subspecialists.

Upon examination, these rashes were consistent with non-blanching, palpable purpura with no tenderness or pruritus. Other than a heart rate of 120 beats/minute, his vitals were normal. He was well oriented to time, place, and person. The patient denied any chest pain, joint aches, headaches, bleeding in urine or stools. Four weeks prior to the presentation, he was started on Bactrim DS thrice weekly since he was on chronic high dose steroids. The patient was compliant with his home medications, including prednisone 30 mg/day orally and home oxygen therapy for idiopathic pulmonary fibrosis (IPF). For gout, he was compliant with colchicine 0.6 mg/day. 

Initial lab workup revealed leukocytosis to 18K with no platelet abnormalities and mild acute kidney injury (AKI) with unremarkable electrolytes. Chest X-ray was consistent with chronic reticulonodular interstitial fibrosis in bilateral lungs with peripheral pleural thickening in bilateral upper lungs and left midlung with no evidence of active disease. Urinalysis was consistent with urinary tract infection (UTI). Taking into account the high leukocyte count and UTI, 2 sets of blood cultures were obtained, which were positive for gram-negative rods. A diagnosis of sepsis secondary to urinary tract infection and superimposed gram-negative bacteremia was established, and intravenous ceftriaxone was initiated. IgA vasculitis was suspected as it is associated with AKI and the palpable purpura.

Prednisone was increased to 40mg daily, and a rheumatologist was consulted. The patient was found to have elevated erythrocyte sedimentation rate (ESR), c-reactive protein (CRP), and low C3 levels. The rheumatologic workup including rheumatoid factor, anti-SSA/anti-SSB, anti-MPO/PR3 and anti-neutrophil antibodies (ANA), hepatitis, and human immunodeficiency virus (HIV) panels were negative. Serum IgA and serum protein electrophoresis were within normal limits. 

To diagnose the purpura, a skin punch biopsy was taken from the left ankle, which confirmed leukocytoclastic vasculitis with IgA deposition in the walls of superficial dermal blood vessels. During the 7-day hospital course, the patient remained afebrile and responded well to treatment. The skin lesions were almost healed at the time of discharge. Repeat blood and urine culture were negative before discharge. Bactrim was permanently discontinued. He was discharged with oral cefpodoxime to complete 14 days of antibiotics and prednisone 40mg/day with a plan for outpatient follow-up in 4 weeks for a urinalysis to screen for any relapse or progression of the condition. The repeat urinalysis during the follow-up visit was normal, with a normal protein-to-creatinine ratio. Continuing regular follow-up of the patient for LCV remission and to check for patient's compliance with medical treatment.

## Discussion

Approach to differential diagnosis

With the initial presentation of rash and AKI, it was at first thought to be secondary to systemic involvement. Elevations in inflammatory markers ESR and CRP suggested an inflammatory process. Provided the patient’s history of IPF, the rash could have been sequelae of underlying autoimmune disorders like IgA vasculitis with systemic involvement, hepatitis C-related cryoglobulinemic vasculitis, drug-induced immune thrombocytopenia, steroid-induced purpura, rheumatoid arthritis (RA), Sjogren syndrome.

Sjogren syndrome was ruled out after a rheumatological blood workup, which depicted the absence of anti-SSA and anti-SSB antibodies (Ab). RA too was ruled out after ANA, anti-citrate citrullinated peptide (CCP) Ab were negative along with the absence of arthralgias. Also considered was corticosteroid-induced purpura, but the distribution of the rash in a gravitational manner provided evidence against this suspicion. 

After ruling out alternative systemic etiologies, primary or secondary leukocytoclastic vasculitis became the working diagnosis. A skin biopsy with histopathology was consistent with a diagnosis of LCV. Considering the patient’s multiple comorbidities, LCV could be a secondary presentation. The underlying cause could be sepsis, or alternatively, Bactrim. As for the pathogenesis of LCV, which usually begins to take effect within 7-28 days after exposure to an inciting agent. The first appearance of the rash, in this case, was 7 days after initiation of Bactrim DS, further suggesting Bactrim as the inciting factor. After various approaches, the diagnosis was finally confirmed to be LCV secondary to Bactrim likely triggered by superimposed acute stress of sepsis secondary to UTI and gram-negative bacteremia.

In retrospect, the patient improved after discontinuation of Bactrim; but our concern here is the development of LCV despite the patient being on longstanding moderate dose corticosteroids therapy.

Pathogenesis

LCV is a condition caused by the unregulated activation of the immune system, including recruitment of leukocytes and activation of the complement cascade. Since it is unregulated, this, in turn, damages the tissues of the body. LCV can sometimes be a part of systemic involvement, and other times it may be the sole presentation of systemic disease. The stimulus is sometimes unknown, which makes it idiopathic or primary LCV, while in the majority of the cases, it is secondary to an inciting event or agent, which most probably would be a drug or an infection [2]. Provided the inciting agent, there is hyperactivation of the immunologic cascade, which most commonly presents in the lower extremities as there is a reduplication of the basement membrane to handle the gravitational pressure. Moreover, there is increased stasis of blood, hemoconcentration and a cooler temperature, all these factors with the activated immune cascade lead to changes of vasculitis [2,3]. 

The deposited immunoglobulins (Ig) activate the immune cascade to release chemokines like interleukins (IL) IL-1, IL-6, IL-8, and tumor necrosis factor (TNF), expression of adhesion molecules, increased stasis, and extravasation of leukocytes, mainly neutrophils. These extravasated neutrophils release reactive oxygen species, collagenases, and elastases leading to fibrinoid necrosis of the vessel wall [4].

Discussion

Vasculitis can involve any vessel in the body; hence the International Chapel Hill Consensus updated the nomenclature in 2012. This nomenclature is based upon the size of the vessel involved and also its etiology (Table 1) [5].

**Table 1:** Nomenclature of vasculitis as per the international Chapel Hill Consensus

Size of the blood vessel involved	Involved entities
Large vessel vasculitis (LVV)	Takayasu arteritis (TAK); giant cell arteritis (GCA)
Medium vessel vasculitis (MVV)	Polyarteritis nodosa (PAN); Kawasaki disease (KD)
Small vessel vasculitis (SVV)	Antineutrophil cytoplasmic antibody (ANCA)–associated vasculitis (AAV); microscopic polyangiitis (MPA); granulomatosis with polyangiitis (Wegener’s) (GPA); eosinophilic granulomatosis with polyangiitis (Churg-Strauss) (EGPA); immune complex SVV; anti-glomerular basement membrane (anti-GBM) disease; cryoglobulinemic vasculitis (CV); IgA vasculitis (Henoch-Schonlein); hypocomplementemic urticarial vasculitis (anti-C1q vasculitis)
Variable vessel vasculitis (VVV)	Behcet’s disease (BD); Cogan’s syndrome (CS)
Single-organ vasculitis (SOV)	Cutaneous leukocytoclastic angiitis; cutaneous arteritis; primary central nervous system vasculitis; isolated aortitis; others
Vasculitis associated with systemic disease	Lupus vasculitis; rheumatoid vasculitis; sarcoid vasculitis; others
Vasculitis associated with probable etiology	Hepatitis C virus-associated cryoglobulinemic vasculitis; hepatitis B virus-associated vasculitis; syphilis-associated aortitis; drug-associated immune complex vasculitis; drug-associated ANCA-associated vasculitis; cancer-associated vasculitis; others

The spectrum of etiologies is widespread for secondary LCV; to date, there is no fixed number of etiologies. We tried summarizing the commonly known ones in Table 2 [6-10].

**Table 2:** Commonly known etiologies of LCV TNF - tumor necrosis factor, LCV - leukocytoclastic vasculitis.

Group of etiology	Individual entity
Infections	Staphylococcus aureus; Neisseria; chlamydia; mycobacterium; human immunodeficiency virus; chronic hepatitis B infection; chronic hepatitis C infection; syphilis
Drugs	Vancomycin; sulfonamides; beta-blockers; erythromycin; beta-lactams; furosemide; thiazides; gold; amiodarone; non-steroidal anti-inflammatory drugs (NSAIDs); TNF-alpha inhibitors; selective serotonin reuptake inhibitors; warfarin; valproate; metformin; allopurinol
Systemic diseases	Sjogren syndrome; systemic lupus erythematosus; inflammatory bowel disease
Tumors	Lymphomas

This list summarizes only the causes that have the potential to trigger LCV. Among the current literature, there have been reported cases with various other causal etiologies. E.g., a case report by Josiah An et al. [7] found dabigatran (a novel anticoagulant) as a cause in a male patient who was being treated for deep vein thrombosis. Another such interesting case was reported by Gupta et al. [8] of levetiracetam leading to LCV in a 14-year-old female. This proves that there is no fixed list of agents that can lead to LCV.

To date, LCV has been a diagnosis of exclusion. Multiple conditions should be ruled out before establishing a diagnosis of drug-induced LCV. To maintain uniformity, the American College of Rheumatology established the following criteria for diagnosis of LCV:

1) Age >16 years at the time of disease onset; 2) medication use and its correlation with disease onset; 3) palpable purpura; 4) maculopapular exanthema; 5) histopathological picture encompassing arterioles and venules with evidence of peri/extravascular granulocytes. The presence of 3 out of the 5 criteria has a diagnostic specificity of 83.9% and sensitivity of 71% [9]

Due to its isolated presentation, involving the blood vessels of the skin exclusively, histopathology is the mainstay to support the diagnosis of LCV. It highlights the immunologic chaos comprising perivascular neutrophils and eosinophils, karyorrhexis of neutrophils, leakage of red blood cells, endothelial cell edema, and fibrinoid necrosis of the vessel wall. Immunofluorescence can also be used to directly visualize the deposited Ig, but the routine implication is debatable since they are present in low quantities in the lesions [10,11].

The case reported here is unique, as the patient was already on chronic steroids, which in theory should have prevented the manifestation of LCV. Notably, after the patient was admitted to the hospital on an increased dose of steroid treatment, the lesions resolved. Does this suggest a specific threshold of steroid dose to be used to treat LCV? Or does it implicate any deficiencies in our knowledge of the pathogenesis of LCV? After having ruled out potential causes, the underlying cause in our case was thought to be Bactrim DS, as the timeline of rash occurrence was congruent with the initiation of Bactrim and resolved after its discontinuation. On the contrary, the patient's presentation was suggestive of an infectious etiology to the LCV, provided the bacteremia secondary to the urinary tract infection. Although taking into account the series of events, the initiation of Bactrim corresponded most clearly with the appearance of purpura on the ankle within one week.

Of concern is the causative agent or rather a component of Bactrim that could potentially amplify the immune response in a way to cause LCV. Sulfur-containing drugs are notorious for immune-mediated reactions like Stevens-Johnson Syndrome (SJS) and type one hypersensitivity reactions, sometimes even leading to anaphylactic shock. Hence, it could be a possible causative component responsible for LCV seen in this patient; however, there is no appropriate evidence explaining the causal relationship at this time. 

The treatment for LCV is primarily centered on immunosuppression or immunomodulation. The effective drugs studied to date are:

1) Steroids 0.5 - 1 mg/kg/day; 2) colchicine 0.5 - 1 mg/kg/day [12]; 3) dapsone 200 - 400mg/day [13]; 4) hydroxychloroquine 200 - 400 mg/day [14]. Colchicine remains under clinical trials for establishing efficacy in preventing LCV relapses [15]. Moreover, chronic colchicine could have lesser tolerability compared to steroids as it can cause gastrointestinal upset. Also, dapsone may not be administered in patients with glucose 6 phosphate dehydrogenase (G6PD) deficiency. Apart from these medications, non-steroidal anti-inflammatory drugs (NSAIDs) can help alleviate the symptoms of pain and urticaria, if present. Supportive measures like leg elevation may also be beneficial.

When LCV is a consequence of systemic disease, or the aforementioned treatments are found ineffective or contraindicated, immunomodulators come into play:

Azathioprine 1 - 2mg/kg/day [16]; methotrexate 0.2 - 0.3 mg/kg/week [17], with folate supplementation; mycophenolate mofetil 2 - 3 gm/day [18] are the immunomodulators being used for treatment of refractory LCV. But, these immunomodulators are a double-edged sword, as they treat the LCV but carry along multiple undesirable systemic side effects.

## Conclusions

The culprit in our case was determined to be Bactrim DS, likely triggered by superimposed acute stress of sepsis secondary to UTI and bacteremia. However, the presentation of LCV in a patient on chronic corticosteroids is rather unique. This case report emphasizes enlisting LCV as one of the differentials in a patient with a similar presentation since, although corticosteroids and colchicine may be the proposed treatment for LCV, they failed to demonstrate their prophylactic potential. Having said that, this case report opens up avenues for the pathological reevaluation of the disease process and pharmacological reconciliation considering the treatment aspect of LCV.
